# Simultaneous alcohol and cannabis use is associated with daily consequences reflective of alcohol use disorder symptoms

**DOI:** 10.1016/j.drugalcdep.2025.112924

**Published:** 2025-10-14

**Authors:** Lindy K. Howe, Olivia L. Bolts, Jane Metrik, Rachel L. Gunn

**Affiliations:** a Center for Alcohol and Addiction Studies, Brown University School of Public Health, Providence, RI 02903, USA; b Alpert Medical School of Brown University, Department of Psychiatry and Human Behavior, Providence, RI, USA; c Providence VA Medical Center, Providence, RI 02908, USA

**Keywords:** AUD, Co-use, Alcohol, Simultaneous use, Alcohol-related consequences

## Abstract

Alcohol use is common in the U.S., with 10.9 % of adults meeting criteria for alcohol use disorder (AUD) in 2023. Co-use of alcohol and cannabis is also widespread and is associated with increased alcohol-related harms. Few studies have examined how different co-use patterns, such as simultaneous (same day use with overlapping effects) versus concurrent (same day use without overlapping effects) use affect alcohol-related consequences, particularly those reflecting AUD symptoms. This study compares the associations between daily simultaneous, concurrent, and alcohol-only use patterns with the likelihood of endorsing alcohol consequences categorized as AUD symptoms. Participants (*N* = 116, 56 % female at birth, M_age_=23.2) completed a 28-day ecological momentary assessment study, reporting daily alcohol and cannabis use and alcohol-related negative consequences. Analyses were preregistered. Consequences were categorized into four AUD categories based on DSM-5 criteria: impaired control, social impairment, risky use, and pharmacological effects. Multilevel binomial logistic regressions assessed the relationship between daily substance use patterns and each AUD consequence subtype. Compared to simultaneous use, concurrent use (i.e. using in the same day but without overlapping effects) was associated with a lower likelihood of endorsing (all p < 0.05): impaired control (OR=0.45), social impairment (OR=0.47), risky use (OR=0.41), and pharmacological effects (OR=0.36). No significant differences were found between simultaneous and alcohol-only days. Results underscore the relevance of the timing and pattern of alcohol and cannabis co-use in understanding alcohol-related problems. Further research is needed to explore the clinical implications of concurrent versus simultaneous use for AUD prevention and treatment.

## 1. Introduction

Alcohol use is widespread in the United States, with 49.6 % of young adults reporting alcohol consumption in the past month and 10.9 % meeting criteria for alcohol use disorder (AUD) in 2023 (Bailey and McHugh, 2023). Co-use of alcohol and cannabis is also common (Lee et al., 2022; Jackson et al., 2020), with growing concern about the potential for cannabis use to exacerbate alcohol-related harms (Bedillion et al., 2024; Gunn et al., 2024; Sokolovsky et al., 2020). Simultaneous use of alcohol and cannabis (i.e., use at the same time such that the effects overlap) is associated with more severe alcohol-related consequences compared to using either substance alone (Lee et al., 2022). Concurrent use (i.e., consuming both substances on the same day without overlapping effects) and alcohol-only use are associated with alcohol-related consequences as well, though simultaneous use is both more common and associated with more consequences relative to concurrent use (Drohan et al., 2023; Subbaraman and Kerr, 2015). However, while studies have examined the association between simultaneous use and alcohol consequences broadly, no studies have focused specifically on daily consequences that reflect the core symptoms of AUD and their association with simultaneous use.

An AUD diagnosis (Diagnostic and Statistical Manual of Mental Disorders 5th ed.; DSM-5; American Psychiatric Association, 2013) includes 11 criteria that reflect a problematic pattern of alcohol use leading to clinically significant impairment or distress. These criteria represent AUD symptoms that are persistent, reflecting a chronic pattern of alcohol use. In contrast, ecological momentary assessment (EMA) studies often examine daily alcohol-related consequences (e.g., nausea, vomiting, social disruptions) that are short-term, real world manifestations of these symptoms. For example, commonly assessed EMA consequences such as “getting into an argument” or “acting rude or obnoxious” (Lee et al., 2017) may reflect the DSM-5 symptom of “social or interpersonal problems caused by the substance” (APA, 2013). However, limited work has examined daily consequences from the lens of AUD symptoms. In the current review of the literature, we use "AUD symptoms" to refer to studies examining criteria based on DSM-5 diagnoses, and "AUD-related consequences" or "alcohol consequences" to refer to the daily manifestations of these symptoms as captured in real-time or day-level EMA data.

While the DSM classifies AUD as a unidimensional construct (MacCoun, 2013), some research emphasizes the importance of considering symptom clusters or presentations as certain AUD symptoms, such as loss of control and social dysfunction, may be associated with unique behavioral and clinical outcomes (Kendler, 2013; Wakefield and Schmitz, 2014, 2015) and harmful externalizing behaviors (McDowell et al., 2019). Prior cross-sectional work has compared specific symptom presentations, such as individuals with withdrawal symptoms versus those without, showing higher likelihood of acute alcohol problems and substance use (Bailey et al., 2021; Howe et al., 2022). However, no EMA studies have examined daily consequences that are related to AUD symptom presentations. Importantly, the DSM-5 further categorizes the 11 AUD criteria into four groups (impaired control, social impairment, risky use, and pharmacological effects) that are suggested to reflect distinct aspects of alcohol-related problems (APA, 2013; McDowell et al., 2019). As symptom groups are thought to phenotypically reflect different problems related to alcohol use (McDowell et al., 2019), examining daily consequences that are reflective of AUD symptom groups may aid in our understanding of the heterogeneous manifestations of AUD.

EMA studies have been instrumental in capturing daily alcohol-related consequences in real time (e.g., nausea, vomiting, social disruptions), demonstrating fluctuations in daily consequences. Further, EMA research has increasingly focused on alcohol and cannabis co-use, investigating the positive association of co-use with alcohol-related outcomes and daily consequences, providing important insight into the role of co-use in conferring risk for alcohol-related problems. For instance, studies suggest that individuals who co-use alcohol and cannabis are at an increased risk of developing AUD (Midanik et al., 2007), and the prevalence of co-use has increased over time (McCabe et al., 2021). Notably, among individuals with AUD, co-use on a given day has been associated with greater likelihood of heavy drinking that day (Metrik et al., 2018). Studies comparing alcohol-only and simultaneous use suggest that simultaneous use is linked to more severe alcohol-related consequences, including impaired cognitive and motor functioning, risky behavior (e.g., driving while impaired), and greater intoxication (Gunn et al., 2022; Lee et al., 2022; Bedillion et al., 2024; Sokolovsky et al., 2020). Prior work also shows that day-level co-use, alcohol-only or cannabis-only days, can differentially impact consequences, such as social conflicts, injury, or hangovers (Drohan et al., 2023; Subbaraman and Kerr, 2015; Wardell et al., 2024).

While recent EMA studies have provided insight into the daily consequences of co-use, prior work has not systematically examined how different patterns of co-use (i.e., simultaneous vs. concurrent) might specifically influence daily consequences. Most EMA studies (i.e., including those reviewed above) have either compared co-use (collapsing simultaneous and concurrent use) versus alcohol-only or focused on simultaneous use versus alcohol-only. While this work is valuable in identifying the positive association between negative consequences and simultaneous alcohol and cannabis use, no prior EMA study has compared all three patterns (e.g., simultaneous, concurrent, and alcohol-only) within the same sample at the daily level. Further, limited studies have examined daily consequences as a way to better understand manifestation of AUD symptoms at the daily level. Although AUD is often considered a chronic condition, symptoms can develop and fluctuate at the daily level, and understanding this variability may provide important insight into the dynamic nature of symptom expression (Dvorak et al., 2014; King et al., 2025). Investigating the relationship between daily co-use and AUD symptom endorsement could offer a more nuanced understanding of how co-use contributes to AUD development and expression, further informing prevention and intervention efforts.

By categorizing daily consequences reported via EMA that are theoretically aligned with DSM-5 AUD groups, this study addresses gaps in the co-use EMA research and allows for a nuanced comparison of simultaneous, concurrent, and alcohol-only use on specific symptom patterns. Additionally, the current analyses are not intended to develop a daily AUD symptom measure, but rather to examine alcohol-related consequences within the context of daily experiences reflective of AUD symptoms. Building on prior evidence that simultaneous use often produces higher levels of overall consequences than concurrent or alcohol-only use (Lee et al., 2022; Wardell et al., 2024; Sokolovsky et al., 2020; Gunn et al., 2022a, 2022b; Subbaraman and Kerr, 2015), the current study examines whether daily patterns of alcohol and cannabis use (specifically simultaneous use compared to concurrent use, and simultaneous use compared to alcohol-only use) are differentially associated with AUD symptom categories. Based on prior work, we hypothesize that: simultaneous alcohol and cannabis use days will be associated with a higher likelihood of experiencing AUD symptom categories than both concurrent and alcohol-only use.

## 2. Methods

### 2.1. Procedures

#### 2.1.1. Recruitment and screening

These analyses are part of a larger study examining patterns and consequences of alcohol and cannabis co-use among young adults. Additional details on study design, procedures, and primary outcomes are reported in Gunn et al. (2024). Participants were recruited through community-based advertisements aimed at young adults who use alcohol and cannabis. These advertisements included flyers and social media posts. Individuals who expressed interest in the study were screened for eligibility via phone or an online questionnaire. Eligible participants were then invited to complete a screening session, where they provided informed consent, confirmed their eligibility through a mental health screener, and completed a Timeline Follow Back interview (Sobell et al., 1979; Sobell et al., 1996) by trained staff.

Inclusion criteria required participants to report consuming alcohol at least twice per week and heavy drinking (i.e., more than four drinks per drinking occasion for self-identified women, more than five drinks per occasion for self-identified men) at least once per week over the last 60 days. Additionally, participants were required to report using cannabis at least once per week on average over the past 60 days and simultaneous use of alcohol and cannabis at least once in the past 30 days. Other eligibility requirements included being aged 18–30 years, fluency in English, being a smartphone user, not currently receiving or seeking treatment for alcohol or cannabis, and absence of suicidal ideation, mania, and psychosis. Full study details and inclusion/exclusion criteria can be found in Gunn et al. (2024). Characteristics of the analytical sample of 116 participants are provided in Table 1.

#### 2.1.2. Baseline and orientation session

Once participants were confirmed to meet eligibility criteria, they completed a baseline session and orientation session. To accommodate university policies related to the COVID-19 pandemic and participant preferences, these orientation sessions were conducted in-person or virtually via Zoom. During the orientation, participants were introduced to the EMA protocol, which included guidance on using the mobile survey application for reporting alcohol and cannabis use and alcohol-related consequences.

#### 2.1.3. Daily surveys

As part of the full EMA protocol (Gunn et al., 2024), participants were prompted to complete daily morning surveys for 28 consecutive days using their smartphones (via Metricwire). Each morning, participants completed a self-initiated report that assessed their alcohol and cannabis use and related consequences from the previous day. Participants received notifications to complete the assessment at 9:00 AM and a reminder at 11:00 AM. Compliance with these morning reports was high, with 88% of all morning surveys being completed.

### 2.2. Measures

#### 2.2.1. Alcohol and cannabis use

Each morning, participants were prompted to recall their substance use from the previous day with the question: “Did you use alcohol and cannabis yesterday so that their effects overlapped?” The available response options included: “Yes, and the effects overlapped,” “I used both alcohol and cannabis, but the effects did not overlap,” “I only used cannabis,” and “I only used alcohol.” Based on their responses, participants were then directed to follow-up questions specific to their reported use. For example, those who indicated they only used alcohol were guided to questions about alcohol quantity and consequences, while those who reported using both alcohol and cannabis were directed to questions about the quantity and consequences of both substances.

Daily substance use patterns were determined based on participants’ responses. If participants reported using both alcohol and cannabis with overlapping effects, their use type was classified as “simultaneous” (event n = 787). If participants used both substances without overlapping effects, their use type was categorized as “concurrent” (event n = 382). If participants reported using only alcohol, their use type was classified as “alcohol only” (event n = 312). Descriptive statistics on person-level averages for each daily substance use pattern type (simultaneous, concurrent, and alcohol only) are provided in Table 1.

#### 2.2.2. AUD-reflecting consequences

A total of 14 negative alcohol-related consequences were assessed each morning for the day prior. Possible consequences are listed in Table 2. Alcohol-related consequences were grouped into symptom clusters based on the DSM-5 criteria for AUD (American Psychological Association, 2013). Specifically, four clusters were derived from consequence items: Impaired Control, Social Impairment, Risky Use, and Pharmacological effects. This approach was theoretically grounded to align with clinically validated AUD criteria and to ensure relevance for capturing functionally meaningful patterns of alcohol-related harm. Due to the zero-inflated distribution of the data, where many participants reported zero occurrences, the consequence variables were dichotomized into "present" (1) and "absent" (0). This approach was chosen to address the skewness in the data and to focus on the presence versus absence of consequences, consistent with prior research in similar contexts.

Impaired Control was represented by the item "Drank more alcohol than originally planned." The Social Impairment cluster included items related to the negative effects of alcohol use on social functioning, such as "Neglected responsibilities," "Got in an argument or fight," "Acted rude, obnoxious, or insulting," and "Said or did embarrassing things." Risky Use encompassed behaviors associated with hazardous alcohol consumption and physical harm, represented by items "Injured self," "Drove car drunk or high," "Blackout," "Passed out," "Had difficulty concentrating," "Felt lethargic or sedated," or "Felt depressed, sad, or anxious”. Pharmacological effects reflected the physiological effects of alcohol use and was represented by the items "Felt nauseous or vomited" and "Hangover." Person-level endorsement rates for each symptom cluster are presented in Table 3.

#### 2.2.3. Covariates

Day-level covariates in the present analyses were weekend (1 =weekend, defined by Thursday-Saturday), total number of drinks consumed, and day in the study (range: 1–28). Person-level covariates included average daily drinks consumed throughout the EMA study, sex assigned at birth (0 =male, 1 =female), age, and self-reported discrimination chronicity to measure social determinants of health in lieu of racial categories (Krieger et al., 2005; Sternthal et al., 2011). In addition, a person-level co-use variable was included to represent the average amount of co-use days for each participant during their EMA reports to control for between-person variation. Similarly, average drinking quantity was included in all models to account for between-person variation in daily drinks, and daily drinks was person-mean centered to represent each participant’s deviation from their own average drinking quantity.

##### Discrimination.

As is standard in research questions related to substance use outcomes, we included a covariate capturing minority group status. We opted to use perceived discrimination rather than race or ethnicity, as it provides a more direct measure of a social determinant of health and a mechanism by which individual differences are associated with substance use outcomes (Krieger et al., 2005; Reeve et al., 2011). Prior research has shown that discrimination is substantially associated with substance use outcomes, making it an important covariate to include in our models. A person-level chronicity subscale of the Everyday Discrimination Scale Short Version (Sternthal et al., 2011), collected at baseline, was included as a person-level covariate of self-reported discrimination chronicity rating (e.g. “In your day to day life how often are you treated with less respect than others” followed by “What do you think the main reason for this?” with options including race). Never was coded as 0, while others were given values based on frequency (e.g., "a few times a year" = 3 times/year). These recoded values were then summed to calculate the total annual experiences of discrimination, with a range from 0 to 575, with higher values representing more chronic experiences of discrimination.

### 2.3. Data management and analysis

Data management and analysis were conducted in R 4.4.1 (R Core Team, 2024). A series of multilevel binomial logistic regressions (GLMMs) were used to evaluate the role of daily simultaneous use on the likelihood of endorsing each AUD-related consequence cluster (i.e., impaired control, social impairment, risky use, and pharmacological effects). Models were specified as follows: Simultaneous use days [Ref] relative to Concurrent use and Alcohol-Only days predicted the likelihood of endorsing each AUD symptom cluster, with a binomial outcome for each (1 =endorsed, 0 =not endorsed) and a random intercept for Subject ID. For all 4 models, analyses controlled for day-level and person-level covariates listed above. Simultaneous use (versus concurrent use and alcohol-only use) was selected as the comparison condition given it was comprised of the greatest number of days relative to other types of days, providing more reliable estimates, and is hypothesized to be associated with the most problems. As such, analyses did not directly compare concurrent vs. alcohol only days. In addition, exploratory post-hoc pairwise comparisons were conducted using estimated marginal means to examine all contrasts among Alcohol-Only, Concurrent, and Simultaneous use days. Missing data was infrequent, with 88% of cases being fully complete. Cases with missing data were excluded using listwise deletion, ensuring that only complete cases were analyzed.

#### 2.3.1. Transparency and openness

The current project is a secondary data analysis of the study outlined in Gunn et al. (2024) and focuses on new predictors and outcomes and investigates unique research questions within the context of the original work. Secondary analyses were registered (https://doi.org/10.17605/OSF.IO/X7TK9). This study was reviewed and approved by the Institutional Review Board of Brown University. Materials and analyses for the original study and this study are available upon request.

## 3. Results

### 3.1. Impaired control

Compared to simultaneous use days, concurrent use days were associated with a lower likelihood of endorsing impaired control (OR=0.45, p = 0.02), while no significant difference was observed between simultaneous use days and alcohol-only use days (OR=0.75, p = 0.44). Regarding covariates, drinking quantity was positively associated with impaired control on a given day (OR=1.31, p < 0.001), indicating that higher daily alcohol consumption was linked to an increased likelihood of endorsing impaired control. At the person-level, the average number of drinks consumed across days (OR=0.60, p = 0.02) was negatively associated with the likelihood of endorsing impaired control, suggesting that individuals with a higher average consumption rate were less likely to experience impaired control. Full model results are displayed in Table 4.

### 3.2. Social impairment

Concurrent use was also associated with a lower likelihood of endorsing social impairment symptoms compared to simultaneous use (OR=0.47, p = 0.02). No significant difference was observed between simultaneous use days and alcohol-only use days (OR=0.91, p = 0.75). Regarding covariates, drinking quantity consumed at the daily level was positively associated with social impairment (OR=1.24, p < 0.001), indicating that higher alcohol consumption on any given day was associated with an increased likelihood of endorsing social impairment. Additionally, a significant positive effect of study day was observed, indicating that participants were more likely to endorse social impairment as the study progressed (OR=1.21, p = 0.04). Finally, a negative relationship was found with age, suggesting that older individuals were less likely to endorse social impairment (OR=0.62, p = 0.02).

### 3.3. Risky use

Concurrent use was associated with a significantly lower likelihood of endorsing risky use compared to simultaneous use (OR=0.41, p < 0.001). However, no significant difference was found between alcohol-only days and simultaneous use (OR=0.69, p = 0.21). Regarding covariates, total drinks consumed on a given day was positively associated with risky use (OR=1.18, p < 0.001), suggesting that higher daily alcohol intake was linked to an increased likelihood of endorsing risky use. No person-level covariates were associated with risky use.

### 3.4. Pharmacological effects

Concurrent use was associated with a lower likelihood of endorsing pharmacological effects compared to simultaneous use (OR=0.36, p < 0.001), while no significant difference was observed between simultaneous use and alcohol-only use days (OR= 0.71, p = 0.28). Regarding covariates, total drinks consumed on a given day was positively associated with the likelihood of endorsing pharmacological effects (OR=1.43, p < 0.001), indicating that higher daily alcohol consumption increased the likelihood of reporting pharmacological effects. Additionally, the average number of drinks consumed at the person-level was negatively associated with endorsing pharmacological effects (OR=0.62, p = 0.01), suggesting that individuals who typically drank more frequently were less likely to endorse this symptom cluster. Finally, a higher discrimination score was positively associated with endorsing pharmacological effects (OR=1.31, p = 0.04), indicating that individuals with greater chronicity of perceived discrimination experiences were more likely to report pharmacological effects such as hangovers or nausea.

### 3.5. Exploratory post-hoc comparisons

Post-hoc pairwise comparisons of estimated marginal means were conducted to evaluate all contrasts among Alcohol-Only, Concurrent, and Simultaneous use days. For all four AUD consequence clusters, results indicated significant differences between Simultaneous and Alcohol-Only days and no significant differences were observed between Simultaneous and Concurrent use days, consistent with primary model findings. In contrast, no significant differences emerged between Alcohol-Only and Concurrent use days. Full tables of pairwise contrasts are provided in the Supplemental Materials.

## 4. Discussion

This study examined the effects of daily simultaneous alcohol and cannabis use on AUD-related consequences among a sample of non-treatment-seeking adults. Findings provide insights into the nuanced effects of simultaneous use on consequences and highlight the relationship between alcohol consumption and AUD-related consequence incidence. For all categories of AUD-related consequences, simultaneous use of alcohol and cannabis is linked to a higher likelihood of all consequence categories (i.e., impaired control [e.g., drinking more alcohol than planned], social impairment [e.g., saying or doing something embarrassing], risky behaviors [e.g., driving drunk, injuring self], and pharmacological effects [e.g., hangover] consequences compared to concurrent use. However, there were no significant differences in AUD-related consequences between simultaneous use and alcohol-only use days, suggesting heightened risks associated with both simultaneous use and alcohol-only use.

Results suggest timing of alcohol and cannabis use may play a role in the presentation of AUD symptoms (notably defined here via daily consequences), consistent with previous studies suggesting that concurrent use (i.e., when substances do not overlap in effect) may result in fewer negative outcomes than simultaneous use (Drohan et al., 2023; Subbaraman and Kerr, 2015). When alcohol and cannabis are consumed at the same time, the combined effects of both substances appear to amplify the severity of AUD-related consequences, also aligning with previous research suggesting that simultaneous use leads to greater intoxication, cognitive and motor impairments, and riskier behaviors (Gunn et al., 2022; Lee et al., 2022). Some theories suggest that cannabis might substitute for alcohol, leading to reduced drinking and fewer alcohol-related consequences. While current results suggest that substitution may occur in the context of concurrent use days, they do not support substitution when cannabis and alcohol are used simultaneously, indicating important nuance in the substitution hypotheses. Post-hoc comparisons suggest that the heightened risk for AUD-related consequences in the current sample is specific to simultaneous use days, as no significant differences were observed between alcohol-only and concurrent use days, highlighting the particular risk associated with simultaneous consumption. Future research should also consider the ordering of alcohol and cannabis use (e.g., alcohol-first vs. cannabis-first days) within simultaneous use, as this may further contextualize the observed effects and reveal whether the sequence of use influences the severity of AUD-related consequences (Gunn et al., 2021). Further, while not explored in the current study, it is possible that the context in which alcohol-only, simultaneous, and concurrent use occur may differ, influencing the outcomes observed (Looby et al., 2021; Gunn et al., 2021; Boyle et al., 2023). Future research should consider how contextual factors, such as social setting or emotional state, shape substance use patterns and related consequences.

Interestingly, findings suggest that alcohol alone carries a similar risk of negative outcomes to those associated with simultaneous alcohol and cannabis use. Findings diverge from previous studies showing that negative consequences from simultaneous use were more pronounced than on alcohol-only (Wardell et al., 2024). At the daily level, it may be that the high levels of alcohol consumption associated with simultaneous use result in consequences comparable to heavy alcohol use rather than substances complementing each other in a way that mitigates risk. For instance, Gunn et al. (2022) found that simultaneous use was associated with a higher drinking rate, but this did not necessarily lead to greater negative consequences compared to alcohol-only use. Further, several mechanisms have been suggested to influence the impact of cannabis on alcohol outcomes, such as individual differences in use patterns, the context of co-use, cannabinoid formulation, and the timing and order of use (Gunn et al., 2022) and should be further explored. Alcohol quantity emerged as a critical covariate in the present findings. Higher daily alcohol consumption was positively linked to all four AUD-consequence symptom categories (impaired control, social impairment, risky use, and pharmacological effects). While not a focal predictor, this underscores the dose-response relationship between alcohol consumption and the severity of AUD symptoms, suggesting that as daily alcohol consumption increases, so does the likelihood of endorsing AUD symptoms across multiple domains (Boyle et al., 2024; Carpenter and Merrill, 2021). This is particularly relevant for a young adult sample as consumption-related DSM-5 criteria are some of the most prevalent AUD criteria within this sample (Bailey et al., 2021; Wardell et al., 2024). It is also worth noting that the inclusion of total drinks in the models may have contributed to the attenuation of some effects, potentially explaining why certain conceptually meaningful differences (e.g., between alcohol-only and simultaneous use days) did not reach statistical significance.

At the person level, individuals who consumed greater average daily alcohol were less likely to report impaired control (i.e., drinking more than planned) and pharmacological effects at the daily level. While seemingly counterintuitive, there are several potential explanations that warrant further exploration. Regarding impaired control, individuals who drink less on average may be more likely to report impaired control at the daily level, as their overall drinking pattern is lower relative to those who drink more heavily (Cooke et al., 2016) and thus may be more likely to experience impaired control on days with heavier consumption. In contrast, individuals who regularly consume larger amounts of alcohol may already plan for higher intake, reducing the likelihood of exceeding their planned consumption and experiencing impaired control (Hamilton et al., 2022). More research on drinking intentions (e.g., planned drinking; Howe and Finn, 2024; Stevens et al., 2022) can help shed light on the relationship between average drinking quantity and experience of impaired control.

Regarding pharmacological effects, this may also be an effect of tolerance, in that individuals who drink more frequently and in higher quantities might have developed a higher tolerance to alcohol (Corbin et al., 2013; Schuckit et al., 2008), making them less likely to experience pharmacological symptoms such as hangovers overall at the person-level. For example, people with higher average alcohol consumption may be less sensitive to the immediate negative effects of alcohol, including impaired control and pharmacological symptoms, due to their greater physiological adaptation to alcohol use. These findings emphasize the importance of tolerance in shaping the experience of AUD symptoms, highlighting the need to consider both day-level and person-level drinking habits when understanding alcohol’s effects.

### 4.1. Study limitations

There are several limitations to consider. First, our sample predominantly comprised White young non-treatment-seeking adults, limiting the generalizability of our findings to diverse in terms of sociodemographic characteristics and clinical samples. Future work should test these findings among a more representative sample, particularly among those in treatment for AUD, to determine whether patterns observed here hold in clinical populations. Further, our findings suggest that perceived discrimination is significantly associated with the pharmacological criteria for AUD, with higher levels of discrimination linked to increased odds of endorsing alcohol-related physiological consequences, such as nausea and hangovers. This underscores the importance of considering the broader social context, including discrimination, in understanding the development and severity of AUD symptoms.

Second, while intensive longitudinal data allows for examining day-to-day effects of substance use, the lack of precise temporal sequencing within the day prevents the ability to draw firm conclusions about causal directionality. For example, withdrawal symptoms often lead to a bidirectional feedback loop. Another limitation of this study is the relatively low endorsement of the negative consequences in the sample. While the clustering of symptoms is a strength in managing low base rates, the low endorsement of negative consequences may limit the ability to detect significant effects. In addition, the present analyses did not control for the potential complexity of the cannabis products used (e.g., THC potency, mode, products). These confounding factors would be important to examine in future studies, as certain formulations, such as high-potency products, are related to higher likelihood of cannabis problems and CUD (Hines et al., 2020), but little work has examined their association with AUD. Finally, outcomes in the current study were assessed as consequences and were categorized based on theoretically grounded DSM-5 symptoms. While other work has begun to theorize daily consequences in this way (King et al., 2025), future work may utilize alternative data-driven models (e.g., Howe et al., 2022; Bailey et al., 2021; Howe et al., 2022; Wakefield and Schmitz, 2015) to confirm the daily representation of AUD symptoms.

### 4.2. Clinical implications and future directions

Results of this study suggest that simultaneous use of cannabis and alcohol increases risk of alcohol consequences, emphasizing the need to target simultaneous alcohol and cannabis use directly in those experiencing alcohol-related problems. Current analyses are preliminary and theory-driven. Future studies explicitly designed to test daily AUD symptoms (e.g., via factor analysis of daily items) will be important to further validate the clinical DSM groupings. Because higher daily alcohol consumption is consistently linked to a greater likelihood of endorsing AUD symptoms, these findings reiterate that interventions focused on reducing daily intake could be a key strategy in mitigating the severity of AUD across multiple domains.

## Supplementary Material

Appendix A

## Figures and Tables

**Table 1:** Sample characteristics (N = 116) and descriptive statistics.

Characteristic	Value	Min.	Max.	Scale Max.
**Baseline Characteristics**				
Sex at birth – Female, n (%) Gender, n (%)	65 (56%)			
Man	49 (42%)			
Woman	58 (50%)			
Genderqueer	1 (<1%)			
Transman	5 (4%)			
Nonbinary	8 (7%)			
Race, n (%)^a^				
American Indian or Alaskan Native	2 (2%)			
Asian American	7 (6%)			
Black or African American	18 (16%)			
Native Hawaiian or Pacific Islander	1 (<1%)			
White (Caucasian)	92 (79%)			
Other^b^	7 (6%)			
Ethnicity – Hispanic or Latino, n (%)	28 (24%)			
Years of Education	14.52 (2.32)	2	18	
Age (years)	23.2 (3.4)	18	30	30
Perceived Discrimination	74.9 (136.2)	0	575	575
AUDIT	12.1 (4.9)	4	27	30
TLFB Alcohol Frequency (60 day)	32.9 (12.9)	13	60	60
TLFB Alcohol Quantity (drinks/event)	4.9 (1.9)	1.9	11.4	
**EMA Substance Use Event Type**				
Simultaneous (event N = 787)	6.7 (5.8)	0	27	28
Concurrent (event N = 387)	3.3 (4.6)	0	21	28
Alcohol-Only (event N = 312)	2.7 (3.9)	0	18	28

Note. Value represents Mean and Standard Deviation (M[SD]) unless otherwise specified. Min = minimum person level average, Max = maximum person level average. TLFB = Timeline Follow Back. Averages of EMA substance use event represent the average number of days a person reported that event type during their time in the study.

a Count accumulates beyond the sample size as participants could select all that applied.

b “Other” race was indicated as “Costa Rican”, “Hispanic”, or “Mixed”

**Table 2:** Consequence items and person-level averages.

Consequence Item	AUD Symptom Subtype	Average Person-Level % Endorsement	Person-level Min %	Person-level Max %
**1. Drank more alcohol than originally planned**	Impaired Control	7.8%	0	57.1%
2. Neglected responsibilities	Social Impairment	9.1%	0	100%
3. Got in an argument or fight	Social Impairment	2.5%	0	33.3%
4. Acted rude obnoxious, or insulting	Social Impairment	1.4%	0	20%
5. Said or did embarrassing things	Social Impairment	6.2%	0	66.7%
6. Injured self	Risky Use	0.2%	0	12.5%
7. Drove car drunk or high	Risky Use	1.8%	0	36.4%
8. Blackout	Risky Use	0.8%	0	25%
9. Passed out	Risky Use	3.2%	0	66.7%
10. Had difficulty concentrating	Risky Use	5.2%	0	50%
11. Felt lethargic or sedated	Risky Use	5.2%	0	45.5%
12. Felt depressed, sad, or anxious	Risky Use	4.0%	0	37.5%
13. Felt nauseous or vomited	Pharmacological Criteria	5.9%	0	58.3%
14. Hangover	Pharmacological Criteria	11.1%	0	75%

Note. Average person-level endorsement reflects % of days where each consequence item was endorsed. Person-level min and person-level max reflect the minimum and maximum % total of days a participant reported that consequence throughout the EMA study.

**Table 3:** AUD symptom subtype descriptives.

Cluster	Average Person-Level % Endorsement	Person-level Min %	Person-level Max %
Impaired Control	7.8%	0%	57.1%
Social Impairment	15.6%	0%	100%
Risky Use	15.0%	0%	86.7%
Pharmacological Criteria	14.3%	0%	75%

Note. Average person-level endorsement reflects % of days where a symptom subtype item was endorsed. Person-level min and person-level max reflect the minimum and maximum % of total days a participant reported a consequence within the respective symptom cluster the EMA study.

**Table 4:** Model results of subtype on use-pattern (Simultaneous, Concurrent, Alcohol-Only).

	Impaired Control			Social Impairment			Risky Use OR (SE)			Pharmacological Criteria		
	OR (SE)	95% CI	p	OR (SE)	95% CI	p	OR (SE)	95% CI	p	OR (SE)	95% CI	p
Day-Level												
Use: Concurrent	**0.45** **(0.35)**	**[0.23, 0.91]**	**0.02**	**0.47 (0.31)**	**[0.26, 0.87]**	**0.02**	**0.41 (0.28)**	**[0.24, 0.71]**	**< .001**	**0.36 (0.32)**	**[0.2, 0.68]**	**< .001**
Use: Alcohol Only	0.74 (0.39)	[0.34, 1.59]	0.44	0.91 (0.3)	[0.51, 1.64]	0.75	0.69 (0.3)	[0.39, 1.23]	0.21	0.71 (0.32)	[0.38, 1.33]	0.28
Total Drinks	**1.31 (0.04)**	**[1.2, 1.42]**	**< .001**	**1.24 (0.04)**	**[1.15, 1.33]**	**< .001**	**1.18 (0.03)**	**[1.11, 1.26]**	**< .001**	**1.43 (0.04)**	**[1.32, 1.54]**	**< .001**
Day of EMA	0.82 (0.11)	[0.67, 1.02]	0.07	**1.2 (0.09)**	**[1.01, 1.44]**	**0.04**	**0.87 (0.08)**	**[0.74, 1.03]**	**0.1**	0.98 (0.09)	[0.82, 1.17]	0.83
Weekend	0.71 (0.23)	[0.45, 1.11]	0.13	0.73 (0.2)	[0.5, 1.07]	0.11	1.25 (0.18)	[0.88, 1.76]	0.21	0.99 (0.2)	[0.67, 1.48]	0.98
Person-Level												
Average Drinks	**0.6 (0.23)**	**[0.38, 0.93]**	**0.02**	0.91 (0.23)	[0.58, 1.42]	0.68	0.75 (0.17)	[0.54, 1.05]	0.1	**0.62 (0.19)**	**[0.43, 0.91]**	**0.01**
Use: Co-Use	0.58 (0.69)	[0.15, 2.23]	0.43	0.72 (0.69)	[0.19, 2.78]	0.64	0.74 (0.55)	[0.25, 2.16]	0.58	0.56 (0.58)	[0.18, 1.72]	0.31
Sex at birth	1.77 (0.39)	[0.82, 3.8]	0.14	0.72 (0.42)	[0.32, 1.64]	0.43	1.09 (0.32)	[0.58, 2.02]	0.8	1.5 (0.34)	[0.77, 2.95]	0.24
Age	1.08 (0.17)	[0.77, 1.52]	0.67	**0.62 (0.19)**	**[0.42, 0.9]**	**0.01**	1.15 (0.14)	[0.87, 1.51]	0.33	**0.5 (0.16)**	**[0.36, 0.68]**	**< .001**
Discrimination	1.11 (0.17)	[0.8, 1.54]	0.54	1.22 (0.19)	[0.84, 1.77]	0.3	1.2 (0.14)	[0.92, 1.59]	0.18	**1.39 (0.14)**	**[1.05, 1.84]**	**0.02**

Note: For “Use”, the reference group is “Simultaneous”. For “Weekend”, the reference group is “weekday”. For Sex at birth, the reference group is “female”. Person-level “Co-use” pattern represents person’s ratio of that event pattern type throughout the study.

## Appendix A. Supporting information

Supplementary data associated with this article can be found in the online version at doi:10.1016/j.drugalcdep.2025.112924.
