# Supplementary material for: Simultaneous alcohol and cannabis use is associated with daily consequences reflective of alcohol use disorder symptoms
Source: Drug Alcohol Depend. Author manuscript; Available in PMC 2026 Mar 26. (PMC13020575; doi:10.1016/j.drugalcdep.2025.112924)
Supplement: Appendix A [file NIHMS2155324-supplement-Appendix_A.docx]

**Supplemental Materials**

Table 1. Pairwise comparisons of differences between the three reference groups

| **Contrast** | **OR** | **SE** | **z-ratio** | **p-value** |
| --- | --- | --- | --- | --- |
| *Model 1: Impaired Control* |  |  |  |  |
| **Simultaneous / Alcohol-Only** | **2.203** | **0.776** | **2.243** | **0.046** |
| Simultaneous / Concurrent | 1.357 | 0.533 | 0.778 | 0.717 |
| Alcohol-Only / Concurrent | 0.616 | 0.293 | -1.019 | 0.565 |
|  |  |  |  |  |
| *Model 2: Social Impairment* |  |  |  |  |
| **Simultaneous / Alcohol-Only** | **2.108** | **0.651** | **2.416** | **0.041** |
| Simultaneous / Concurrent | 1.098 | 0.330 | 0.312 | 0.948 |
| Alcohol-Only / Concurrent | 0.521 | 0.196 | -1.736 | 0.192 |
|  |  |  |  |  |
| *Model 3: Risky Use* |  |  |  |  |
| **Simultaneous / Alcohol-Only** | **2.434** | **0.681** | **3.182** | **0.004** |
| Simultaneous / Concurrent | 1.449 | 0.430 | 1.251 | 0.423 |
| Alcohol-Only / Concurrent | 0.595 | 0.218 | -1.416 | 0.333 |
|  | |  |  |  |
| *Model 4: Pharmacological Criteria* | |  |  |  |
| **Simultaneous / Alcohol-Only** | **2.741** | **0.873** | **3.168** | **0.004** |
| Simultaneous / Concurrent | 1.411 | 0.451 | 1.077 | 0.528 |
| Alcohol-Only / Concurrent | 0.515 | 0.213 | -1.607 | 0.243 |

Note. Bolded categories represent statistically significant effects.
